# Identifying predictors of patient safety competency based on sleep quality in student faculty of nursing and midwifery during the internship period: a multidisciplinary study

**DOI:** 10.1186/s12912-024-01725-2

**Published:** 2024-01-24

**Authors:** Mohammad Javad Jafari, Pouya Mostafazadeh, Mohammad Reza Mojebi, Reza Nemati-Vakilabad, Alireza Mirzaei

**Affiliations:** 1 Students Research Committee, School of Nursing and Midwifery, Ardabil University of Medical Sciences, Ardabil, Iran; 2 Student Research Committee, School of Nursing and Midwifery, Guilan University of Medical Sciences, Rasht, Iran; 3 Department of Emergency Nursing, School of Nursing and Midwifery, Ardabil University of Medical Sciences, Ardabil, Iran

**Keywords:** Nursing education, Healthcare students, Internship, Patient safety, Patient safety competency, Sleep quality

## Abstract

**Background:**

Ensuring patient safety is of paramount importance in healthcare services. Sleep disorders not only have detrimental effects on the health of healthcare students but also significantly impair their performance, leading to an increased risk of medication errors. These errors can pose a grave threat to the safety and well-being of patients. It is crucial to address and mitigate sleep disorders among internship healthcare students to safeguard the quality of care and minimize potential patient harm.

**Objectives:**

This study aimed to investigate the predictors of Patient Safety Competency (PSC) based on the sleep quality of internship healthcare students.

**Methods:**

A study was conducted on 331 students from the Ardabil School of Nursing and Midwifery at Ardabil University of Medical Sciences in northwest Iran from August to December 2022. The participants were selected by stratified random sampling. Data were collected using a demographic information form, the Pittsburgh Sleep Quality Index (PSQI), and the Health Professional Education in Patient Safety Survey (H-PEPSS). The collected data were analyzed using SPSS software version 22.0. Person correlation coefficients were used to examine the relationship between PSC level, its dimensions, and sleep quality, while multiple linear regression was conducted to identify the predictors of PSC.

**Results:**

The competency of nurses in patient safety was average in both classroom and clinical settings. However, their ability to work as a team with other healthcare professionals scored the lowest. In addition, the quality of sleep was found to be a predictor of patient safety competency among healthcare students during their internships.

**Conclusions:**

It is important to note that healthcare students tend to have moderate patient safety competence (PSC), which is positively correlated with their sleep quality. Therefore, it is vital to identify the key factors that directly affect PSC. This would enable nursing and midwifery faculty administrators to take preventive measures to enhance patient safety competence in both classroom and clinical settings. Additionally, organizing educational workshops that engage students and improve their sleep quality could improve patient care. Practical courses are recommended for health professionals and students in clinical settings to enhance patient safety competencies. Additionally, student internships should receive hands-on training to improve teamwork and rest conditions.

## Introduction

Patient safety (PS) is a crucial aspect of healthcare systems as it indicates the quality of patient care. It involves preventing and reducing adverse events that may harm patients during their care and mitigating their potential consequences [1]. In developed countries, one out of every ten patients experience harm due to errors and side effects during their hospital stay [2]. In the United States, medical errors account for more than 250,000 deaths per year, making it the third leading cause of death. For this reason, patient safety disease has been highlighted as one of the main challenges because, in many cases, patient care - in addition to system failure, weak organizational processes and faulty management - also requires health professionals [3]. Therefore, patient safety should be the fundamental priority of healthcare providers, and they should have a higher level of knowledge about patient safety [2]. There is a consensus among healthcare professionals that students pursuing healthcare education must acquire essential knowledge, skills, and tools to prevent errors and adverse events by the time they complete their university education [4]. Additionally, healthcare students need to receive adequate education on sleep health to ensure that they are prepared to provide safe and effective patient care [5].

Healthcare students need to receive proper training to become competent healthcare providers. Colleges have been urged to prepare students for clinical environments so they can provide safe and appropriate care, which will improve patient safety competency (PSC) [6]. Healthcare professionals are responsible for providing primary care, coordinating patient care, educating patients and their families, and providing information [7]. PSC refers to the necessary abilities to perform the role of a healthcare provider and includes knowledge, attitude, and clinical skills [1]. Understanding the competencies of healthcare workers is critical to improving their preparation as crucial healthcare team members [8]. According to the Canadian Patient Safety Institute (CPSI) definition, PSC consists of six domains: patient safety culture, effective communication, safety risk management, optimization of human and environmental factors, identification and reporting of adverse events, and teamwork [9, 10]. Undergraduate nursing education is an important starting point in promoting PSC regarding knowledge, attitude, and skills to prepare the health team members for the future [11]. Providing PS skills to healthcare students is highly important, especially in the classroom and clinical settings, and it significantly affects their behavior and clinical skills [10]. However, high-stress levels experienced during schooling and lack of sleep can negatively affect healthcare students’ academic performance and physical and mental health [12].

Sleep is integral to cognitive functioning, physiological tasks, and emotional control, which is necessary to maintain energy and restore body function [13]. For various reasons, healthcare students often experience poor sleep quality. Inadequate sleep with insufficient quality and quantity leads to fatigue, poor performance, higher safety risks, decreased cognitive performance, and unstable mood in students, which can affect PSC [14]. According to a study conducted in Mashhad, Iran, approximately 30% of medical students reported having sleep problems [15]. Additionally, Kaur et al. said that 43.5% of students experience poor sleep quality due to excessive workload and irregular working hours [16]. A systematic review reported that healthcare providers commit most medical errors due to high workloads, insufficient night sleep, and too much stress [17]. The inability of healthcare students to maintain optimal sleep quality can lead to poor PSC, characterized by poor confidence in their knowledge of PS [18].

Orem’s self-care deficit nursing theory (2001) points to the ability of individuals to perform activities to maintain life, health, and well-being and to participate in self-care activities. Self-care consists of adequate rest that causes human growth [19]. However, healthcare students often neglect their self-care [20]. Due to high academic requirements and stressful situations during their education, healthcare students often experience poor sleep quality.

Iran’s Ministry of Health and Medical Education has developed accreditation standards for hospitals to ensure PS and has required healthcare centers to implement PS criteria [21]. In recent years, the effective inclusion of the components of safety culture, PS issues, and competencies needed to maintain PS in the educational programs of various healthcare professions has been emphasized to promote PS [22]. Providing safe care requires PSC. Hence, such competencies should be integrated into professional health education that prepares healthcare providers [1].

Previous studies have mainly focused on investigating the link between sleep quality and patient safety skills in healthcare students. While the impact of sleep deprivation has been a critical area of interest, it is essential also to explore how various aspects of sleep quality can affect patient safety competencies, especially among internship healthcare students in Iran. Therefore, conducting a study to examine the association between sleep quality and patient safety skills among students at Ardabil Nursing and Midwifery Faculty can help fill this research gap.

## Methods

A cross-sectional, descriptive-analytical study was conducted on students at Ardabil University of Medical Sciences in northwest Iran from August to December 2022.

### Setting and sample

The Ardabil School of Nursing and Midwifery offers undergraduate degrees in nursing, emergency medicine, midwifery, and operating room studies. The program typically lasts for four years, and students begin their clinical internships in the second semester. During the clinical internship course, each student is paired with a mentor who provides guidance and support starting from the second semester. Currently, 471 students are undergoing clinical training in these areas. Only students in the third semester or later who have started their clinical internships are eligible for inclusion in this study. First-year students are excluded from the study as they need to complete clinical training during the preliminary year. The Ardabil University of Medical Sciences Ethics Committee has approved this study for Internal Review Board (IRB) purposes.

The study aimed to ascertain the sample size based on a confidence level of 99% and a margin of error of 0.05%. The Epi Info StatCalc program suggested 275 students to achieve the required sample size. After considering a non-response rate of 20%, the estimated final sample size was 331. The study used a stratified random sampling method, with the sample size for each field determined by the number of students in that field. Specifically, the sample size for nursing was 221 students, midwifery was 45 students, the operating room was 35 students, and emergency medicine was 30 students. The questionnaires were distributed randomly among the students. The researcher will keep the data in a secure file cabinet in their office for one year after the study ends.

### Data collection

After the study details were explained, the students were provided with written informed consent forms to sign. Only those who met the inclusion criteria were requested to complete a set of paper questionnaires in their classrooms. Participation in the study was voluntary, and students who agreed to participate were asked to sign the informed consent forms. The paper-based questionnaires were then distributed among the students, and it took approximately 15 min to collect the data.

### Instruments

The data was collected using three different forms: a demographic information form, the Pittsburgh Sleep Quality Index (PSQI), and the Health Professional Education in Patient Safety Survey (H-PEPSS).

#### Demographic information form

The demographic information form contained 12 items that asked about age, gender, semester, participation in a safety course, and reports of observing or experiencing medical errors.

#### Health professional education in patient safety survey (H-PEPSS)

The second part included a modified version of the Health Professional Education in Patient Safety Survey (H-PEPSS) designed by Ginsburg et al. (2012) [23]. This tool can be used by healthcare professionals, students, and recent graduates as a self-assessment tool for their PS competency.

The H-PEPSS consists of 38 items that assess PSC in three sections. The first section deals with “teaching specific aspects of PS” (27 items). These cases are divided into seven dimensions: (1) Clinical safety issues (4 items), (2) Socio-cultural domains (6 items), (3) Effective communication (3 items), (4) Safety risk management (3 items), (5) Human and environmental factors (3 items), (6) Recognizing and responding to adverse events (4 items), and (7) Safety culture (4 items). In this part, the participants are first asked to rate each item on a Likert scale ranging from strongly disagree (1) to strongly agree (5) regarding what they learned in the classroom and during their clinical experience. The second part of the H-PEPSS consists of seven items that address “how broader issues of PS are addressed in professional health education”. The third section of the H-PEPSS consists of 4 items that assess “talking comfortably about PS”. The second and third parts are scored on a Likert scale from 1 (strongly disagree) to 5 (strongly agree). In the data analysis, the scores for the second and third sections (broader aspects of PS and talking comfortably about PS) are divided into two categories: agree/strongly agree (4 and 5) and neutral/disagree/strongly disagree. This questionnaire has already been translated and validated in Iran [1, 22]. Ten faculty members from Ardabil University of Medical Sciences were invited to review a questionnaire to assess its content validity. The experts evaluated each question based on three criteria: simplicity, appropriateness, and certainty, using a four-part spectrum ranging from relatively simple to complex. The content validity index was calculated for each question and found 0.78.

Additionally, the content validity ratio was calculated to be 0.89. The Cronbach’s alpha coefficient of the questionnaire has been reported to range from 0.73 (clinical safety) to 0.86 (effective communication and safety risk management). The overall reliability of the tool has been reported to be 0.89 [22]. In this study, Cronbach’s alpha coefficient of the questionnaire ranged from 0.83 (clinical safety) to 0.84 (effective communication and safety risk management), and the overall reliability of the tool was 0.94.

#### Pittsburgh sleep quality index (PSQI)

This questionnaire measures sleep quality and disorders during the past month [24]. It contains 19 items and seven subscales, including subjective sleep quality (SSQ), sleep latency (SL), Sleep Duration (SDU), habitual sleep efficiency (HSE), sleep disorders (SD), use of sleep medications (USM), and daytime dysfunction (DD). Each question is rated using a 4-point Likert scale (0–3), from 0 (better) to 3 (worse). The scores of the seven components are then calculated by summing them to create a global PSQI score. The range of scores is from 0 to 21. A score above 6 indicates poor sleep quality. The validity and reliability of this questionnaire have been investigated in Iran, with Cronbach’s alpha of 0.81 [25]. In this study, Cronbach’s alpha coefficient of PSQI was 0.83.

### Data analysis

The data was analyzed using SPSS software version 22.0 (SPSS Inc., Chicago, IL, USA). Various descriptive statistics, including number, percentage, mean, and standard deviation, were utilized to examine the PSC level and its dimensions, sleep quality and dimensions, and students’ demographic characteristics. Pearson’s correlation coefficient was also used to determine the correlation between patient safety competency and sleep quality and its dimensions. Additionally, the relationship between patient safety competency and demographic characteristics was investigated using an independent-sample t-test, One-way ANOVA, and Pearson’s correlation coefficient. The one-sample Kolmogorov-Smirnov test was performed before the statistical tests to ensure the normality of data distribution. Lastly, multiple linear regression was used to analyze the factors that predict participants’ PSC.

### Ethical considerations

The study was conducted under the code IR.ARUMS.REC.1401.231 was approved by the Ethics Committee of Ardabil University of Medical Sciences. The researchers received permission from the vice-chancellor of the Faculty of Nursing and Midwifery to attend students’ classrooms. They introduced themselves to the students and explained the study’s goals, methods, and procedures. The research followed the Helsinki guidelines, and participation was voluntary. The participants were informed of their right to withdraw at any stage if they did not wish to continue. Ethical considerations, such as written informed consent, confidentiality, and anonymity, were also taken into account.

## Results

The mean age of the participants was 23.58 ± 2.70 years, and most were male (54.1%). Most students were studying in the 3rd to 6th semester, and most (66.8%) were studying nursing. Further, 135 students (40.8%) stated they had previous PS education experiences. Most healthcare students (57.1%) could not participate in the courses previously held in the college, hospital, or virtual spaces. Moreover, 191 students had a history of reporting medical errors. Also, most participants (54.1%) stated that the university education program did not adequately cover PS issues. Most students (46.2%) mentioned their level of PSC as average. Some educational characteristics of healthcare students participating in this study are presented in Table 1.

**Table 1:** Demographic characteristics of the participants (*n* = 331)

Variable	Category	Mean (SD)	
Age		22.58 (2.70)	
Duration of PS course		1.11 (1.99)	
		**No.**	**Percentage**
Gender	Male	179	54.1
	Female	152	45.9
Semester	3.00	89	26.9
	4.00	54	16.3
	5.00	59	17.8
	6.00	65	19.6
	7.00	24	7.3
	8.00	40	12.1
Prior experience on PS	Yes	135 196	40.8
education	No		59.2
Patient safety	Virtual Course	25	7.6
course venue	Faculty	109	32.9
	Hospital	8	2.4
	others	189	57.1
Experience or observe adverse events	Yes	278	84.0
	No	53	16.0
Report medical errors	Yes	191	57.7
	No	140	42.3
Adequate coverage of PS issues in the curriculum	Yes	152	45.9
	No	179	54.1
Self-assessment of PS competence	Very Weak	5	1.5
	Weak	32	9.7
	Moderate	153	46.2
	Good	124	37.5
	Very Good	17	5.1
Major	Nursing	221	66.8
	Midwifery	45	13.6
	Surgical Technologist	35	10.6
	Medical Emergency	30	9.1

The dimensions of PSC in the classroom and clinical environments and the dimensions of sleep quality are shown in Table 2. The mean scores of all dimensions of PS in the classroom and clinical environments were higher than 3.61 (out of 5). Students were confident in their learning dimensions of “clinical safety skills” in the classroom (4.01 ± 0.95) and safety culture in the clinical environment (3.99 ± 0.73). Moreover, students had the least confidence in learning “teamwork with other health professionals” in the classroom (3.62 ± 0.77) and recognizing and responding to adverse events in clinical settings (3.83 ± 0.93). There was a statistically significant difference between the mean scores of PS dimensions, except for clinical safety skills, effective communication, and safety culture in the classroom and clinical environment. The statistically significant difference shows that healthcare students’ learning in clinical environments was better than in the classroom. The mean score of the sleep quality index in students was 6.34 (SD = 2.70, range = 0 to 21). Among the sleep quality index dimensions, the highest score was reported for SL and HSE, and the lowest score was found for the dimension of the USM.

**Table 2:** Descriptive statistics (*n* = 331)

Variable		Mean	SD	Min	Max	Range
**PS total in the classroom**		3.79	0.53	2.20	5.43	1–5
Clinical safety skills		4.01	0.95	1.00	5.00	1–5
Work in teams with other health professionals		3.62	0.77	1.00	5.00	1–5
Communicating effectively		3.83	0.76	1.00	5.00	1–5
Managing safety risks		3.76	0.76	1.00	5.00	1–5
Understanding human & environmental factors		3.69	0.77	1.00	5.00	1–5
Recognize and respond to adverse events		3.65	0.67	1.75	5.00	1–5
Culture of Safety		3.96	0.55	2.50	5.00	1–5
**PS total in the clinical practice**		3.91	0.56	1.62	6.19	1–5
Clinical safety skills		3.97	0.80	1.00	5.00	1–5
Work in teams with other health professionals		3.92	0.69	1.67	5.00	1–5
Communicating effectively		3.89	0.77	1.00	5.00	1–5
Managing safety risks		3.93	0.68	1.00	5.00	1–5
Understanding human & environmental factors		3.84	0.69	1.00	5.00	1–5
Recognize and respond to adverse events		3.83	0.93	1.00	5.00	1–5
Culture of Safety		3.99	0.73	1.00	5.00	1–5
**Sleep quality total PSQI**		6.34	2.70	0.00	15.00	0–21
Subjective sleep quality		1.00	0.83	0.00	3.00	0–3
Sleep latency		1.25	0.81	0.00	3.00	0–3
Sleep duration		0.75	0.90	0.00	3.00	0–3
Sleep efficiency		1.17	1.35	0.00	3.00	0–3
Sleep disturbances		1.07	0.60	0.00	3.00	0–3
Use of sleep medication		0.31	0.63	0.00	3.00	0–3
Daytime dysfunction		0.76	0.82	0.00	3.00	0–3

Pearson’s correlation coefficient showed a statistically significant positive correlation between the total score of patient safety competency in the classroom and sleep quality dimensions. However, it had a statistically significantly inverse relationship with the “sleep efficiency” subscale. The total score of the PSC had a statistically significant positive relationship with the dimensions of sleep quality, except for the HSE and SDU dimensions. In other words, the PSC score increased (*P* < 0.05) with an increase in the sleep quality dimension score. No statistically significant correlations were observed between the broader aspects of PS and dimensions of sleep quality, except for the USM dimension. Also, no statistically significant correlation was found between talking comfortably about PS and sleep quality dimensions, except for the USM dimension (Table 3).

**Table 3:** Relationship between internship healthcare students perceived confidence relating to PS dimensions and sleep quality subscales (*n* = 331)

Variable	1	2	3	4	5	6	7	8	9	10	11
1. PS total in the classroom	1.000										
2. PS total in the clinical practice	0.675**	1.000									
3. Broader aspects of PS	0.252**	0.502**	1.000								
4. Comfort in speaking up about PS	0.058	0.217**	0.513**	1.000							
5. Subjective sleep quality	− 0.476**	-0.341**	-0.088	0.078	1.000						
6. Sleep latency	-0.347**	-0.186**	0.017	0.0104	0.455**	1.000					
7. Sleep duration	-0.249**	-0.084	0.003	0.070	0.249**	0.194**	1.000				
8. Sleep efficiency	0.132*	0.073	0.073	-0.066	-0.272**	-0.272**	-0.530**	1.000			
9. Sleep disturbances	-0.364**	-0.312**	-0.105	0.001	0.652**	0.303**	0.095	-0.113**	1.000		
10. Use of sleep medication	-0.129*	-0.166**	-0.159**	-0.116*	0.284**	-0.008	0.032	0.059	0.273**	1.000	
11. Daytime dysfunction	-0.289**	-0.283**	-0.014	0.019	0.469**	0.054	0.117*	-0.104	0.382**	0.473**	1.000

**Correlation is significant at the 0.01 level (2-tailed)

*Correlation is significant at the 0.05 level (2-tailed)

The results of multiple regression analysis and predictors of students’ perceived confidence about PS dimensions are shown in Table 4. In four models, multivariate regression analyses were performed using students’ perceived confidence about PS dimensions as dependent variables, demographic characteristics, and sleep quality dimensions as independent variables. In the first model and PSC scale in the classroom, from 18 independent variables, SSQ variables, SL, SDU, SD, DD, academic semester, adequate coverage of PS issues in the curriculum, and self-assessment of PSC accounted for 43% of the variance of the final model (F = 13.42, *p* < 0.001, R^2^ = 0.43). In the second model, the predictors of clinical p.a. PSC include SL, SDU, DD, age, previous experience in PS training, venue of patient safety workshop, medical error report, adequate coverage of PS issues in the curriculum, and self-assessment of PSC, which could account for 37% of the variance of the final model (F = 10.29, *p* < 0.001, R^2^ = 0.37). In the third model, the variables of USM, DD, previous experience in PS training, place of PS workshop, and duration of PS workshop were introduced as the predictors of broader aspects of PS, which were able to account for 15% of the variance of the final model (F = 3.26, *p* < 0.001, R^2^ = 0.15). Finally, the results of multiple regression indicated that the variables of SDU, USM, previous experience in PS education, venue of PS workshop, reporting of medical errors, and adequate coverage of PS issues in the curriculum predicted talking comfortably about PS, which accounted for 12% of the variance of the final model (F = 2.55, *p* < 0.001, R^2^ = 0.12).

**Table 4:** Factors influencing internship healthcare students perceived confidence relating to PS dimension based on sleep quality subscales and demographic characteristics (*n* = 331)

variables	**In the classroom (model 1)**		In the clinical practice (model 2)		Broader aspects of PS (model 3)		Comfort in speaking up (Model 4)	
	Beta	*p*-value	Beta	*p*-value	Beta	*p*-value	Beta	*p*-value
Constant		*p* < 0.001		*p* < 0.001		*p* < 0.001		*p* < 0.001
Subjective sleep quality	-0.180	0.014	-0.001	0.993	0.074	0.407	0.077	0.396
Sleep latency	-0.170	0.001	-0.134	0.015	0.047	0.462	0.022	0.736
Sleep duration	-0.123	0.031	0.027	0.656	0.052	0.456	0.140	0.048
Sleep efficiency	-0.103	0.068	-0.050	0.405	0.097	0.159	0.008	0.905
Sleep disturbances	-0.208	0.001	-0.289	*p* < 0.001	-0.163	0.038	-0.044	0.582
Use of sleep medication	0.091	0.081	-0.016	0.777	-0.172	0.007	-0.168	0.010
Daytime dysfunction	-0.154	0.005	-0.159	0.006	0.088	0.191	0.039	0.565
Age	-0.037	0.402	-0.131	0.005	0.020	0.715	0.053	0.332
Gender	0.011	0.818	0.017	0.734	-0.059	0.313	-0.058	0.333
Semester	-0.201	0.000	-0.049	0.374	-0.032	0.619	-0.049	0.445
Prior experience on PS education	-0.099	0.185	-0.399	P < 0.001	-0.298	0.001	-0.231	0.013
Patient safety course venue	-0.059	0.445	-0.410	P < 0.001	-0.205	0.030	-0.312	0.001
Experience or observe adverse events	0.051	0.329	-0.032	0.562	-0.071	0.272	-0.036	0.582
Report medical errors	-0.086	0.106	-0.207	*p* < 0.001	0.087	0.183	0.160	0.016
Adequate coverage of PS issues in the Curriculum	-0.181	0.000	-0.149	0.005	0.007	0.907	-0.176	0.005
Duration of PS course	-0.033	0.555	0.056	0.341	0.223	0.001	0.015	0.829
Self-assessment of PS competence	0.208	0.000	0.149	0.004	0.084	0.158	-0.009	0.881
Major	-0.036	0.425	-0.035	0.460	-0.060	0.275	-0.018	0.744
	F = 13.42, *p* < 0.001, R^2^ = 0.43		F = 10.29, *p* < 0.001, R^2^ = 0.37		F = 3.26, *p* < 0.001, R^2^ = 0.15		F = 2.55, *p* < 0.001, R^2^ = 0.12	

## Discussion

Sleep disorder not only causes health problems for an internship but also leads to decreased performance and increased incidence of medication errors, which can endanger patient safety. Based on our knowledge, this is the first study investigating the relationship between patient safety competency (PSC) and sleep quality in nursing and midwifery students in Iran. Therefore, this study investigated the relationship between nursing and midwifery students’ perceived trust in patient safety competency and sleep quality.

The results showed the nurses’ PSC in the classroom and clinical settings was average, consistent with previous studies’ outcomes [26]. A study in Iran showed that PSC among nurses was at a moderate level [27]. This finding shows the importance of empowering healthcare students to perform safe clinical procedures to improve PSC. It also suggests adopting a PS framework in the healthcare student’s curriculum in classrooms and clinical environments to improve the PSC of medical students.

The overall mean score was higher in the clinical environment than in the classroom, which is consistent with the results of previous studies [28] and shows that opportunities have been provided for learners to use theoretical lessons. Classroom learning in clinical settings can increase confidence in acquiring PSC [23]. Some researchers believe proper training in PS skills is necessary to clarify roles, functions, and responsibilities and can be a cost-effective solution to prevent adverse patient events [22]. The curriculum has been documented as facilitating the learning of PSC in the classroom. Although there is still uncertainty about how PS can be considered and observed in theoretical and clinical healthcare education (including nursing), evidence suggests that early engagement of healthcare students with PS principles in the classroom can have a significant impact on the development of knowledge, skills, and behaviors related to PS in the long term. Since patient safety education primarily occurs in the classroom, designing and implementing a coherent and robust curriculum for PS seems essential [29].

Regarding PSC in 9 dimensions, teamwork with other healthcare professionals scored the lowest. This result was in line with the findings of previous studies [9, 12, 30]. Based on the results of Alquwez et al., inefficient interprofessional collaboration methods can endanger patients’ safety and their lives [31]. Bressan et al. stated that today, due to the lack of time caused by the increase in the number of nursing students in the classroom, students are not exposed to direct training to learn teamwork with other healthcare professionals in the field of PS [32]. In Iran, there is no medical curriculum on teamwork teaching. Therefore, the uncertainty of this study sample in acquiring sufficient knowledge and competency in teamwork with other health professions can be justified [9]. Given the changing nature of healthcare and the gap between health professions, students must receive teamwork training because Interprofessional Education (IPE) can improve patient care [33].

The results of this study showed that 71% of students had an average sleep quality above five and an average PSQI score of 6.34 (2.7), indicating that most had poor sleep quality [34]. Marta et al. showed that poor sleep quality in nursing students can be attributed to various factors. For example, late-night studying, prolonged Internet use, stress from clinical situations, and lectures/examinations induce poor sleep quality [34]. A study on 7,626 students in the USA showed that 61.9% had poor sleep quality, with a mean score of 6.87 on the PSQI scale. (3.29) [35]. The present study’s findings differed from that of Becker et al. [36]. The different results are due to factors affecting sleep quality, including socio-economic status, academic success, academic background, and general health among students.

Our study showed that mental sleep quality in students was related to PSC in the classroom, consistent with the results of Park et al.‘s study [37]. Sleep disorders and poor SSQ, especially in students, can decrease sleep quality and reduce their productivity and performance in PSC [30]. Poor SSQ is believed to be related to fatigue and burnout [38]. Poor SSQ and SD may significantly affect work performance and successively threaten PS. Therefore, it shows the importance of considering the improvement of SSQ in the design of intervention programs.

The results also showed that SL was related to PSC in the classroom and clinical settings. Extended SL and inefficient sleep lead to reduced PSC. Sleepiness during the day may reduce concentration during speaking and lecturing among nursing students and thus reduce their energy and motivation for effective learning [12]. Also, according to the results of Epstein et al., sleep problems and fatigue are common among nursing students and may have consequences for both PS and the nurses’ health [39].

SDU significantly correlated with PSC in the classroom and talking comfortably about PS. The findings of Stimpfel et al. showed that students with lower SDU significantly reported lower PSC, which is in line with the results of this study [40]. In addition, studies have shown that one of the most important reasons is the use of digital tools during the night, which disrupts daily functions, including feeling sleepy in classes, reduced concentration, and tired during the day, which confirms the results of our study [41].

Furthermore, there was a significant relationship between sleep disorders and PSC in the classroom, clinical settings, and broader aspects of PS. The nursing responsibilities, patient care, and the stress caused by facing acute conditions lead to sleep disorders and affect the quality of sleep [42, 43]. Poor sleep quality causes fatigue and is a serious issue because it affects the decision-making of the medical staff and, thus, PS [44]. Inadequate sleep was also associated with poor health among nurses [45]. It can be concluded that good sleep quality is essential for medical students to increase their health and work performance.

DD was one of the factors affecting PSC in the classroom and clinical settings. Decreased sleepiness-induced DD was associated with increased confidence in understanding personal and environmental elements and recognizing and responding to danger. The negative impact of insufficient sleep on students’ knowledge, memory, and performance has been reported previously. Hudson et al. said that reduced SL and improved HSE were associated with students’ increased confidence in their knowledge of PS [46]. Seoane et al. also indicated that short SDU leads to daytime sleepiness among medical students, which is associated with reduced attention and ultimately leads to poor academic performance [47].

The results indicated a relationship between the academic semester and PSC; as the academic semester increased, the PSC decreased, which was in line with the findings of previous studies [10, 31, 48, 49]. The downward trend in students’ confidence in their knowledge about PS as the academic semester progresses may be due to the emphasis and foci of the courses offered each year [12]. Kılıç HF’s results contrasted with our study’s [50]. The results of this study indicated that senior nursing students spend more time in clinics learning how to communicate with patients and work with other health professionals, which in turn increases PSC.

Evidence shows that adequate coverage of PS issues in the curriculum predicts PSC in the classroom and clinic and in talking comfortably about PS. Turkman et al. reported that the educational intervention significantly affected the overall PS competencies of internship healthcare students. Despite the importance of PS and the recommendations provided, there is no effective and regular educational program about PS in nursing schools [51]. Therefore, it is necessary to include PSC in the curricula of undergraduate healthcare students to prepare them for their future roles in clinical practice [52, 53].

Self-assessment of PSC predicted PSC in the classroom and clinical settings, aligning with the results of Huang et al.‘s study [54]. The results of this study indicated that self-education and theoretical learning about PS issues and having a “very good” self-assessment of PSC were essential facilitators of PSC [54]. Suppose we want to improve PS and create harm-free patient environments significantly. In that case, it is necessary to develop effective teaching and learning strategies to properly equip nursing students with sufficient knowledge and skills related to PS to deal with adverse events [55].

Our results showed a significant correlation between age and PSC in clinical settings. The positive correlation between age and PSC supports this finding [50]. Moreover, according to their results, Rebeschi et al. stated that older nursing students have more experience in PS-related issues [56]. From the results of this study, it can be seen that the USM, previous experience in PS education, and the venue of PS courses were predictors of broader aspects of PS issues and talking comfortably about PS, which is in line with the findings of previous studies [22, 56, 57]. Moreover, previous experience in PS training and holding PS courses had a significant relationship with PSC in the hospital. A study conducted in Brazil on sleep quality among medical students showed that students had poorer sleep quality in the early years of university than in the older years due to the USM [57]. The findings of previous studies also indicate that students who use sleep medications also use other sleep stimulants, which negatively affects their sleeping and waking cycles [58]. In addition, having previous experience in PS training can have a significant impact on PSC. Kim et al.‘s quasi-experimental study on the effects of a PS course using a flipped classroom model showed that students’ PSC increased after completing the course [59].

In this regard, Lee et al. investigated the effectiveness of an independent PS course by comparing the PSC, attitudes, and knowledge before and after the completion of the undergraduate course by nursing students and between those who participated and those who did not participate in the course. The results showed that patient PSC was significantly higher in those who participated in the period after the intervention [52]. It is necessary to explain the results of previous studies about the effect of the venue of patient safety courses on PSC are consistent with our findings. Rabeschi stated nursing students who obtained knowledge of PS from the clinical environment felt more confident about PS than those who received wisdom from the classroom [56]. It seems that the type of universities and hospitals where students spend their clinical courses, due to the quality of facilities, coherent training programs, and learning opportunities they provide to their students, influence their effectiveness in obtaining PSC, which may lead to different performance and results in students’ learning. Therefore, more studies are needed to provide more precise evidence about the positive impact on students’ PSC.

Concern about the consequences of reporting medical errors among students is one of the constant challenges in safety-related research, which was significantly related to PSC in clinical environments and talking about PS in the present study [54]. Kilic et al. reported that participants who were aware of the pathways to follow after committing medical errors scored higher on PSC [50]. Therefore, Vaismoradi et al. stated that reporting a medical mistake increases students’ self-confidence, which helps them learn a helpful experience related to that error [60]. However, the study results by Levine et al. differed from our findings [61]. Based on these findings, it can be concluded that if serious measures are taken in PSC about reporting errors and sharing them with other students and instructors, they can prevent PS threats [60].

## Limitations

When interpreting the results of this study, it is important to consider its limitations. Using a self-report questionnaire to assess Patient Safety Culture (PSC) in healthcare students during an internship may not be the most effective method. Future studies should consider using qualitative methods for a more comprehensive analysis. Additionally, longitudinal and controlled studies may be necessary to determine the effectiveness of PS culture and PSC, rather than the cross-sectional design employed in this research. It is important to note that cross-sectional studies can have limited generalizability due to sampling bias, but researchers attempted to minimize this effect by using random sampling. Since this study was conducted in a specific geographical location, its findings may not apply to internship healthcare students in universities in other regions or countries. Therefore, cross-cultural studies are necessary in the future to evaluate these factors in different cultures. Furthermore, it is crucial to investigate factors beyond PSC or lack of sleep when medical errors occur among healthcare students.

## Conclusions

Internship healthcare students must assess their sleeping habits as a component of their overall health. Poor sleep quality can negatively impact the PSC of internship healthcare students in various ways. Therefore, additional research is needed to explore the factors influencing the sleep quality of healthcare students. Appropriate strategies should be implemented to enhance their sleep quality, maintain it optimally, and promote PSC. Nursing curricula can incorporate topics related to sleep and sleep disorders. Providing students with information about the correlation between sleep quality and PSC can ensure patients receive quality and safe care. Further research is required to determine if these findings are consistent across different healthcare students’ programs and countries.

## Data Availability

The data that support the findings of this study are available from the corresponding author [A.M.] upon request.
